# A Novel Typing Method for *Streptococcus pneumoniae* Using Selected Surface Proteins

**DOI:** 10.3389/fmicb.2016.00420

**Published:** 2016-03-31

**Authors:** Arnau Domenech, Javier Moreno, Carmen Ardanuy, Josefina Liñares, Adela G. de la Campa, Antonio J. Martin-Galiano

**Affiliations:** ^1^Servicio de Microbiología, Hospital Universitari de Bellvitge, Universitat de Barcelona, IDIBELLBarcelona, Spain; ^2^CIBER de Enfermedades RespiratoriasMadrid, Spain; ^3^Bacterial Genetics, Centro Nacional de Microbiología, Instituto de Salud Carlos IIIMajadahonda, Spain; ^4^Presidencia, Consejo Superior de Investigaciones CientíficasMadrid, Spain

**Keywords:** diagnosis, emergent clones, genomics, surface proteins, virulence factors

## Abstract

The diverse pneumococcal diseases are associated with different pneumococcal lineages, or clonal complexes. Nevertheless, intra-clonal genomic variability, which influences pathogenicity, has been reported for surface virulence factors. These factors constitute the communication interface between the pathogen and its host and their corresponding genes are subjected to strong selective pressures affecting functionality and immunogenicity. First, the presence and allelic dispersion of 97 outer protein families were screened in 19 complete pneumococcal genomes. Seventeen families were deemed variable and were then examined in 216 draft genomes. This procedure allowed the generation of binary vectors with 17 positions and the classification of strains into surfotypes. They represent the outer protein subsets with the highest inter-strain discriminative power. A total of 116 non-redundant surfotypes were identified. Those sharing a critical number of common protein features were hierarchically clustered into 18 surfogroups. Most clonal complexes with comparable epidemiological characteristics belonged to the same or similar surfogroups. However, the very large CC156 clonal complex was dispersed over several surfogroups. In order to establish a relationship between surfogroup and pathogenicity, the surfotypes of 95 clinical isolates with different serogroup/serotype combinations were analyzed. We found a significant correlation between surfogroup and type of pathogenic behavior (primary invasive, opportunistic invasive, and non-invasive). We conclude that the virulent behavior of *S. pneumoniae* is related to the activity of collections of, rather than individual, surface virulence factors. Since surfotypes evolve faster than MLSTs and directly reflect virulence potential, this novel typing protocol is appropriate for the identification of emerging clones.

## Introduction

*Streptococcus pneumoniae*, the pneumococcus, is a prevalent member of the commensal flora of the nasopharynx. This bacterium can turn into a versatile pathogen with the ability to successfully colonize many environments inside the host (Bogaert et al., 2004). Pneumococcus is a major etiological agent of pneumonia, meningitis, sepsis, and otitis media. The chance of suffering a pneumococcal infection is dependent on the age group, lifestyle, and patient co-morbidities. Different types of disease, symptom severity, and antimicrobial resistance rates associate epidemiologically to different pneumococcal lineages. Thus, a rational classification of isolates would improve patient management. Up to 96 serotypes have been classified according to the immunogenic properties of the polysaccharide capsule. The capsule is an important virulence factor that prevents complement-mediated phagocytosis. However, isolates that have switched their serotype by capsule gene exchange are favored under the selective pressure exerted by the serotype-based vaccines (Brueggemann et al., 2007).

Multilocus Sequence Typing (MLST; Maiden et al., 1998) is a typing method which provides a simplified view of genotypes. It is based on the allelic profiles of seven housekeeping gene fragments (*aroE, gdh, gki, recP, spi, xpt*, and *ddl*), which render sequence types (ST) grouped into clonal complexes (CC). For instance, ST180 and ST181 share all but one allele. Then, ST181 is a single locus variant of ST180. Both STs are grouped into clonal complex CC180, considering ST180 as the founder. However, intra-clonal variability associated with clinical behavior, e.g., local outbreaks, does exist (Silva et al., 2006; Moschioni et al., 2013). Subclones can emerge either from point mutations, deletions/duplications of key genes, or prophage integrations. However, the major source of evolution in *S. pneumoniae* is genetic recombination, a process facilitated by the natural competence of this bacterium. Recently, the massive sequencing of complete genomes has allowed the analysis of recent variations in alternative genes or genomic accessory regions (Donkor et al., 2012; Browall et al., 2013), which were not detectable by MLST or serotyping. These intra-clonal polymorphisms commonly occur on surface proteins (Croucher et al., 2011; Browall et al., 2013), which constitute the communication interface between pathogen and host. Many of these proteins play a role in virulence (Bergmann and Hammerschmidt, 2006). They typically have modular architectures: a universal cell-wall anchoring domain fused to an outer region that determines functional specificity. This outer region can diverge from strain to strain. This sequence divergence dictates surface protein activity and immunogenicity (Gravekamp et al., 1997). Moreover, “Non-Classical Surface Proteins” (NCSP) have also been reported, such as central metabolism enzymes that exert moonlighting activities when located in the cell wall (Bergmann et al., 2001).

Since isolates that have the same MLST may convey different surface proteins that affect pathogenicity, a new postgenomic typing system is required. In this study, we have developed such a system, termed surfotyping.

## Materials and methods

### Family selection

The 19 genomes that were analyzed were selected among the 25 complete closed sequences stored at the NCBI FTP site (status: Jan/2014; Supplementary File S1). Surface proteins were identified using profiles and the Pfam domain search function applying gathering thresholds (Finn et al., 2014): choline-binding proteins (CBPs) using PF01473 and LPxTG-anchor proteins using PF00746. Lipoproteins were predicted with PRED-LIPO (Bagos et al., 2008). NCSPs were obtained from two literature reviews (Bergmann and Hammerschmidt, 2006; Pérez-Dorado et al., 2012).

### Computational surfotype and MLST assignation

Surface proteins were identified in draft proteomes by BLAST using representative protein sequences (Supplementary File S2). BLAST was used using thresholds selected from the gold standard of 19 genomes. The combination of identity and BLAST score thresholds (Supplementary File S3) were established in the average point between lowest *bona fide* hits and the highest non-specific hits. Using the existence or absence of BLAST hits, surfotypes were derived as Boolean vectors. A BLAST *p*-value < 0.001 was required in all cases. Draft genomes were typed by MLST using BLAST. Query sequences used were those of the alleles present in the MLST web page (http://pubmlst.org/spneumoniae/). Assignment of an allele required a 100% identity over 100% length of the sequence. Subsequent ST and CC assignment was carried out using the information available in the same web page. Draft proteomes were downloaded from the public NCBI ftp site, ftp://ftp.ncbi.nlm.nih.gov/genomes/Bacteria_DRAFT/ (Status 15/09/2014; Supplementary File S4).

### Surfotype clustering into surfogroups

The Inter-Surfomic distance (ISD) parameter between two surfotypes (*v* and *w*) was defined as:

$$\begin{array}{l} ISD\left(v,w\right)=\frac{-100}{Log10\overset{17}{\underset{i=1}{\prod }}F_{feat}} \end{array}$$

where *i* stands for protein family index and *F*_*feat*_ stands for the global frequency of the feature (either presence/absence or full/truncated allele) in the dataset of 19 reference genomes if *v* and *w* features matched or a value of 1 if they mismatch. Surfotypes were hierarchically clustered by their *ISD*s using the ward method of the *hclust* procedure available in the fast cluster package (Müllner, 2013) of the R-project.

Cluster feature consistency was calculated for every protein as the percentage of cases that match the most prominent feature in the surfogroup, considering the one with the lowest general frequency in case of a tie. Given that protein features have different occurrence, a normalized consistency (*NC*) for every protein was applied:

$$\begin{array}{l} NC=\frac{\overset{Tj}{\underset{j=1}{\sum }}\left(\frac{n}{v}\right)\times Fn_{j}}{Tj} \end{array}$$

where *j* stands for the cluster index, *Tj* for the total number of clusters, *n* for the total number of surfotypes considered, *v* for the number of features per protein (*v* = 2 in this work) and *Fn*_*j*_ for the natural frequency for the most prominent feature in the cluster *j*. Theoretically, *NC* may range from 50% (all features are equally represented in all clusters) to 100% (just one kind of feature is represented in every cluster).

### Experimental surfotype assignation by PCR

The 17 genes were screened by PCR in 95 isolates collected from patient attended between 2009 and 2011 at the Hospital Universitari de Bellvitge. These 95 isolates were selected as representatives of the different genotype-serotype combinations. MSLT and serotypes were obtained retrospectively from frozen stocks of the isolates. Data was routinely obtained as part of the hospital daily practice. To estimate the relationship between surfogroup and epidemiological data, we assumed that all isolates of each serotype-genotype combination share the same surfogroup. We only considered the 27 cases in which ≥4 clinical records were available for isolates with the same SG-ST combination. Invasive rates and average patient age of clonal complexes were calculated from 610 clinical isolates collected from non-invasive (acute exacerbation of COPD *n* = 131, and non-bacteriemic pneumoniae *n* = 167) and invasive (*n* = 334) pneumococcal disease. Oligonucleotide sequences were acquired from the literature when dedicated papers for the family were available. Otherwise, they were designed for optimal selectivity using the reference genomes on gene regions identical in all family members. PCR conditions and oligonucleotides utilized in this work are listed in Supplementary File S5. Surfotype profiles were assigned to the pre-existing surfogroup with the most significant *p*-value (when < 0.05). The *p*-value was calculated as the product of the probabilities of the matching features between the profile and the surfogroup signature. Unassigned profiles likewise were screened to the surfotype library, but applying a *p*-value threshold of 0.01. The classification performance of all cases (*n*) was quantified through several estimators using true positives (TP), true negatives (TN), false positives (FP), and false negatives (FN). Accuracy was defined as (TP + TN)/n; sensitivity as TP/(TP + FN); specificity as TN/(TN + FP); and precision as TP/(TP + FP).

## Results

### Selection of variable protein families

**Figure 1:**
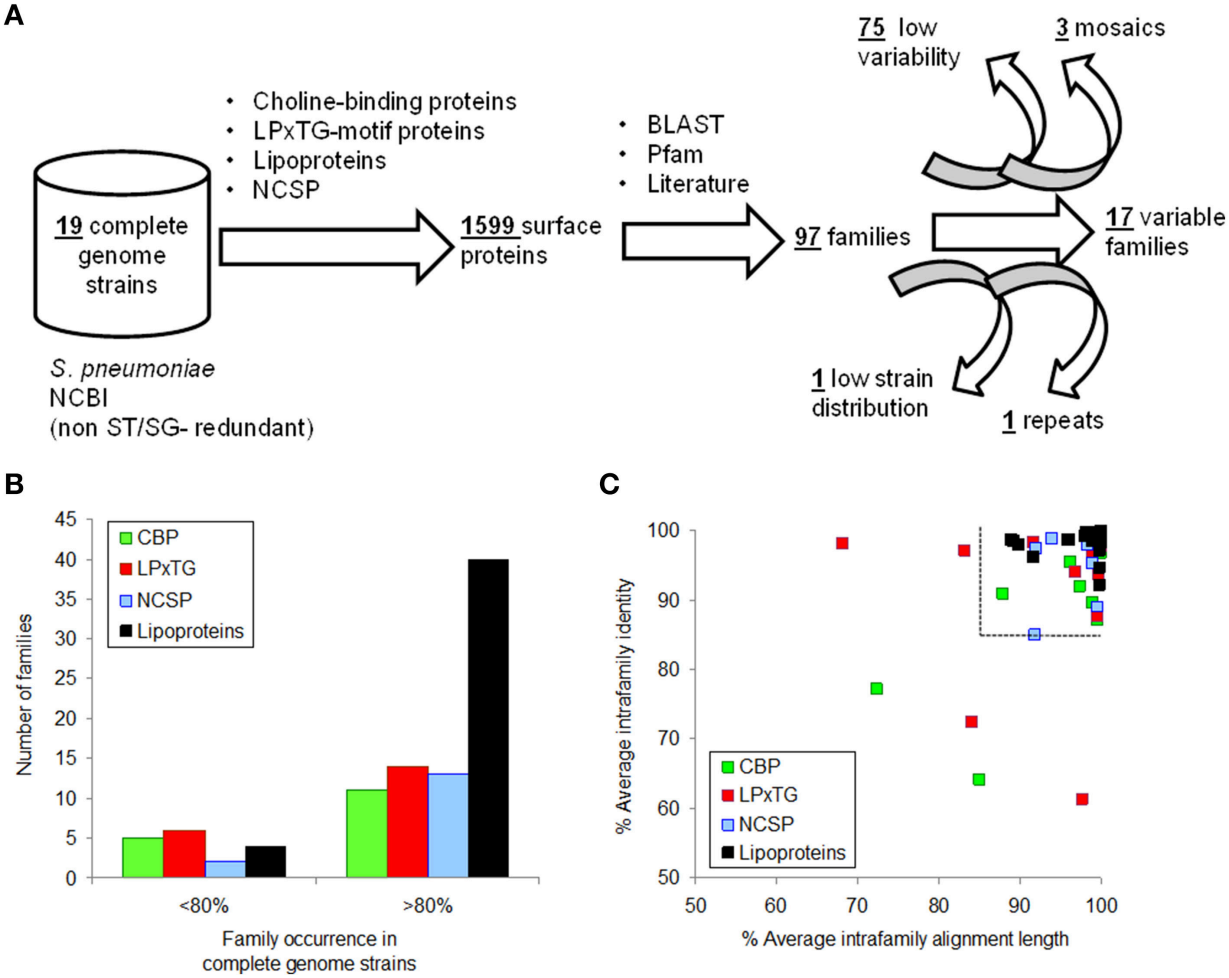
**Selection of surface proteins showing inter-strain variability. (A)** Procedure flowchart used to detect variable proteins. **(B)** Occurrence distribution of protein families according to surface anchor. **(C)** Identity and alignment length averages of families with an occurrence>80% in the reference genomes. The dashed lines split protein families not selected as a consequence of low variability.

Surface proteins showing the highest variability were identified using a gold standard of 19 complete genomes with different surfogroup-sequence type (SG-ST) combinations (Figure 1A). Despite only 11 out of the 96 pneumococcal serotypes are included in these reference genomes, these serotypes are associated to a vast majority of clinical cases. In addition, they carry the virulence factors described in the literature at molecular and clinical detail (Bergmann and Hammerschmidt, 2006; Pérez-Dorado et al., 2012). Proteins considered for further analysis were those containing either a choline-binding domain; the LPxTG domain; the lipoprotein “lipobox” motif; or being reported as NCSP. The 1599 sequences found in the 19 genomes belonged to 97 homolog families (Supplementary File S6). Up to 75 families were present in most reference strains (≥16) and their homologs shared a high identity (≥85%) over most of the sequence alignment (≥85%). These families were considered invariable and were discarded from the analysis (Figures 1B,C). The remaining 22 families showed five kinds of disparity: presence versus absence; full versus truncated versions; continuum of number of repeated motifs; high sequence divergence; and domain mosaicism. Five protein families were further rejected. PavB was rejected because the actual number of repeats can be changed due to genome misassembling (Jensch et al., 2010). CbpA, Iga1, and PspA were rejected because their large sequence divergence or mosaicism (Hollingshead et al., 2000; Iannelli et al., 2002; Bek-Thomsen et al., 2012) prevented the direct comparison between variants. Lrp was rejected because was present in just two strains. Finally, 17 families were chosen for typing (Table 1): 15 with a pattern of presence/absence and 2 with a pattern of full/truncation. Many of these proteins are well-documented virulence factors and show particular Pfam domain combinations.

**Table 1:** **List of selected surface proteins**.

**Symbol^a^**	**TIGR4^b^**	**Description**	**Pfam architecture^c^**
CbpF (CBP)	SP0391	Choline binding protein F	[CB]6
CbpG (CBP)	SP0390	Choline binding protein G	Tripsin-[CB]3
CbpI (CBP)	SP0069	Choline binding protein I	[CB]6
CbpJ (CBP)	SP0378	Choline binding protein J	[CB]8
CbpL(CBP)	SP0667	Choline binding protein L	Excalibur-[CB]7-Lipoprotein_Ltp
DiiA (GPA)	SP1992	Dimorphic invasion-involved protein A	[B02864]1-2-DUF1542-LPxTG
NanC (NC)	SP1326	Neuraminidase C	Sialidase-BNR-BNR_2
NanE (LPP)	SP1330	N-acetylmannosamine-6-P epimerase	NanE
PclA (GPA)	NF	Collagen-like surface-anchored protein	YSIRK-G5-[Collagen]6
PhtA (NC)	SP1175	Pneumococcal histidine triad protein A	[Strep_His_triad]2-B01076-Strep_His_triad
PspC2 (GPA)	NF	Pneumococcal surface protein C	< YSIRK>-RICH- < B16622>- < B9758}>- < [B503]1-2>-LPxTG
PsrP (GPA)	SP1772	Pneumococcal serine-rich	[B214]10-LPxTG
RrgB (GPA)	SP0463	Ancillary pilus subunit B	Cna_B-LPxTG
SP_1796(LPP)	SP1796	Unknown substrate ABC transporter	SBP_bac_1
SrtD (LPP)	SP0468	Sortase D	Sortase
ZmpC (GPA)	SP0071	Zinc metalloproteinase	YSIRK-B134-B5460-B1438-G5-Peptidase_M26_N-B1656 Peptidase_M26_C
ZmpD (GPA)	NF	Zinc metalloproteinase	B5200-LPxTG-G5

a *Protein class. CBP, Choline-binding protein; GPA, Gram-positive anchor (LPxTG motif-containing) protein; LPP, lipoprotein; NC, Non-classical surface protein*.

b *NF, not found in TIGR4 strain*.

c *PfamA and PfamB (those starting by “B”) domains are in sequential order. Accessory domains are in angle brackets. Repeated motifs are in square brackets together with the observed number of repeats. CB, Choline-binding motif. LPxTG, Gram-positive anchor containing the “LPxTG” sortase motif. Pfam domains with the lowest E-values were prioritized. PfamB domains (Eval < 0.01) were also considered only if overlapped < 50% in length with more significant domains*.

### Construction of surfotypes and clustering into surfogroups

Binary patterns for the set of 17 protein families, denoting their presence/absence or full/truncated versions, may reflect the virulent capacity of clones. Representative protein sequences of every family were used to generate a library. The family members from TIGR4 and R6 strains were preferentially chosen since these isolates have been extensively used to study the molecular virulence of pneumococcus. This library was used to perform a BLAST screening on 216 draft proteomes, which covered 110 known STs (and 21 new) grouped into 31 CCs (plus 19 singletons). A total of unique 116 combinations, called surfotypes, were detected.

The convergence between surfotypes was quantified by the ISD (see Section Materials and Methods), a parameter that also considers the relative occurrence, in the dataset, of each protein feature. An ISD matrix between all unique surfotypes was calculated and then subjected to hierarchical clustering. The resulting clades were validated at progressive levels of granularity, from 1 to 40 clusters, calculating feature NC and clonal complex homogeneity at every level (Figure S1). This allows assessing the similarity between surfotype members.

**Figure 2:**
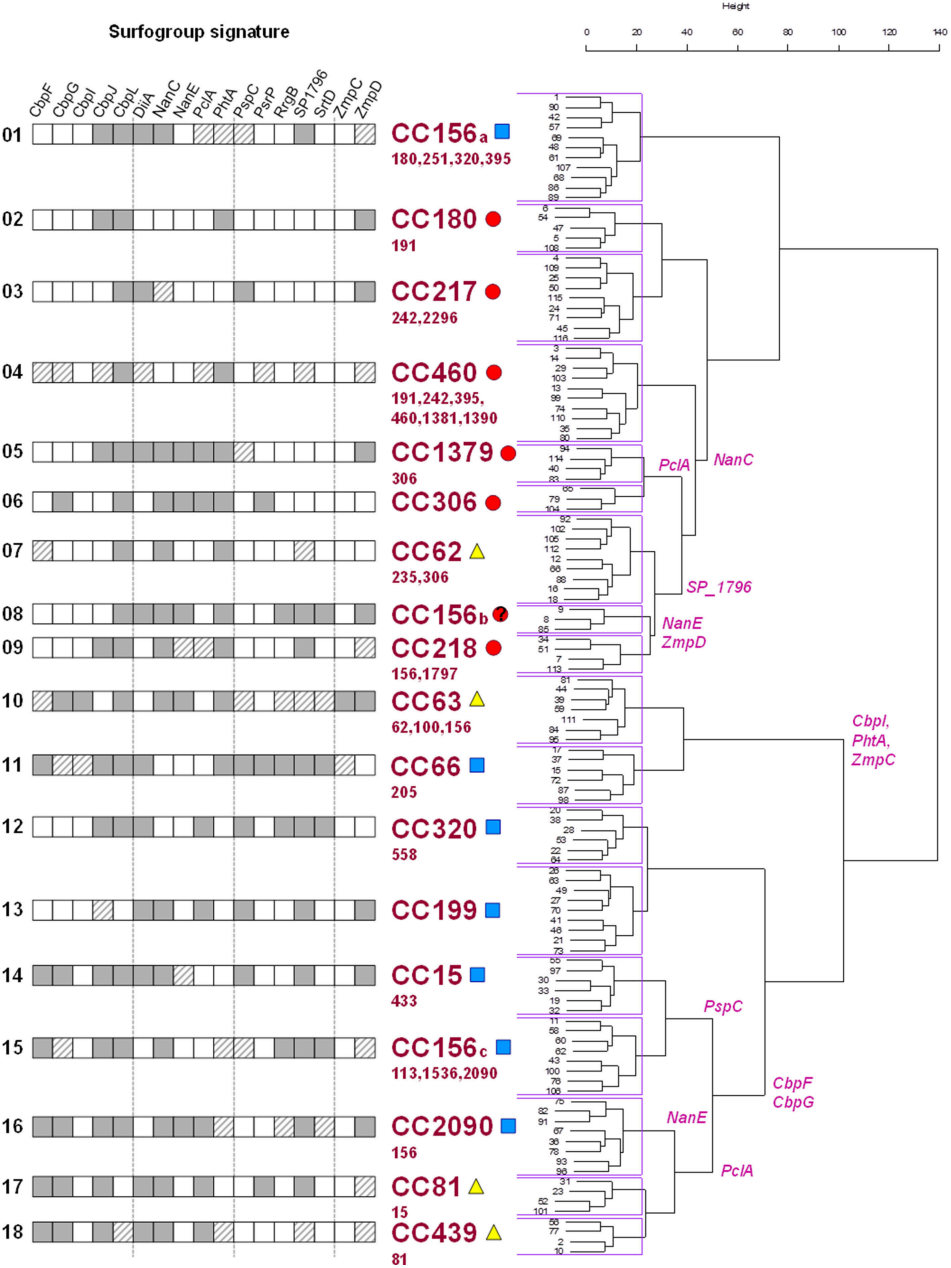
**Hierarchical clustering of surfotypes and correlation to clinical behavior**. Surfogroup signature cells: dark gray (presence/full feature match homogeneity >60%); white (absence/truncated feature match homogeneity >60%); dashed (match homogeneity < 60%). The surfogroup clades are labeled with the most abundant clonal complex together with pathogenic tendency: primary invasive (red circles), opportunistic invasive (blue squares), and non-invasive (yellow triangles). Minority clonal complexes are listed in smaller font size below. Specific protein families responsible from branching (>80% surfotypes in a branch, < 20% surfotypes in the other) are labeled in the tree.

The quality estimators reached an asymptote with 18 clusters, i.e., 87.5 and 67.6% for intracluster NC and MLST clonal complex homogeneity, respectively. From this point, a lower number of clusters caused spurious isolate cross-classification whereas a higher number results in excessive data partitioning without a substantial increment of cluster purity. These meaningful clusters were termed “surfogroups,” whose members shared a minimal common set of protein attributes that were termed “signatures” (Figure 2). The resultant surfogroups were dominated by clonal complexes whose pathogenic behavior is documented in the literature (Supplementary File S7). We utilized the fact that all surfogroups were dominated by a CC. Only if published data concerning the representative CC were scarce or inexistent, virulence was supported by data from its commonest serotype or data from secondary (less abundant) CCs in the surfogroup. Primary invasive were those showing high invasive rates (CC217 and CC306 of serotype 1, 6, and CC191 of serotype 7F) or extreme rates or mortality (CC180 of serotype 3) in young adults. Opportunistic invasive show higher carriage rates, although are still invasive in an age/comorbidity dependent manner. This is typical of CCs linked to 19A and 19F serotypes. Finally, non-invasive can cause non-invasive infection (described above) and/or show high carriage rates (CC81 linked to 23F serotype).

These reported epidemiological data are congruent with the hierarchical tree: seven surfogroups were ascribed to primary invasive (highly invasive in healthy population) isolates, seven were ascribed to opportunistic invasive pneumococcal disease (invasive potential in elderly patients and/or with co-morbidities) and 4 were correlated with non-invasive types of the disease.

### Correlation between surfotyping and MLST

Up to 88 and 96% of isolates with the same ST shared the same surfotype or surfogroup, respectively. The clonal complexes had a more dispersed pattern since only 62 and 84% of strains with the same CC were classified into the same surfotype and surfogroup, respectively (Figure S2A). To obtain further insight into this intra-clonal discrepancy, the analysis was selectively performed on the five most prominent STs (≥5 strains) and CCs (≥10 strains). These STs were variable at the level of the preferred surfotype (37–86%), but essentially belonged to the same surfogroup (Figure S2B). All these CCs contained 5–7 surfotypes from 1 to 2 surfogroups (Figure S2C), with the exception of CC156, which dispersed into 17 surfotypes and 6 surfogroups.

### Surfotyping of clinical isolates

**Figure 3:**
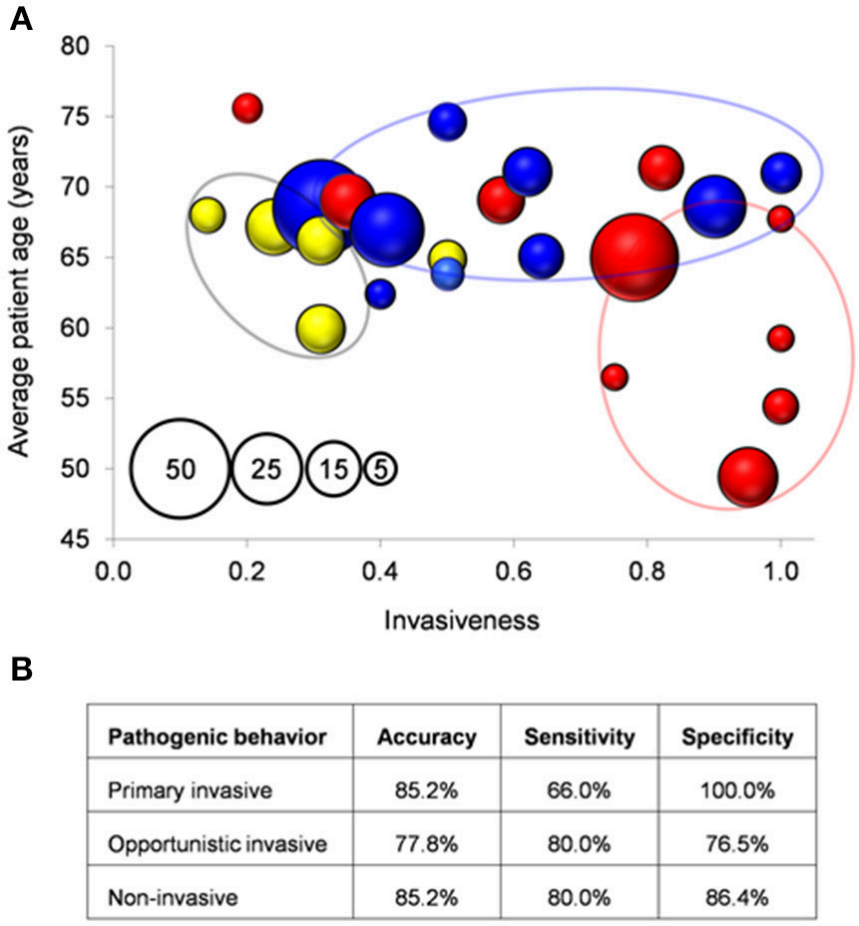
**Correlation between surfogroup and clinical isolates**. **(A)** Each bubble represents a unique SG-ST combination. Bubble size (see pattern in the inset): number of clinical isolates. Bubbles are colored according to type of pathogenicity after surfogroup prediction according to Figure 2. **(B)** Measures of the classification performance.

The 17 genetic features were screened by PCR in 95 isolates showing different genotype-serotype combinations (Supplementary File S8). All the isolates but one (98.9%) were reliably assigned to a surfogroup. To correlate surfogroup and epidemiological data, clinical reports recorded were utilized (See Section Materials and Methods; Figure 3A). The rate of primary invasive predictions was higher for those isolates that were, in fact, invasive (as defined as the ratio of invasive samples in the ST-SG combination) ≥0.75 and patient age ≤ 68 years. Opportunistic invasive predictions mainly appeared in the area of the graph covering an invasiveness score of 0.32–0.75 and an invasiveness score of >0.75 combined with patient age >68 years. Finally, non-invasive predictions correlate with isolates with an invasiveness score of < 0.32. Using these clinical boundaries, surfogrouping predicted correctly 20 out of 27 tested ST-SG combinations (precision = 74.1%, *p*-value = 0.006 Fisher's exact test; Figure 3B).

## Discussion

**Figure 4:**
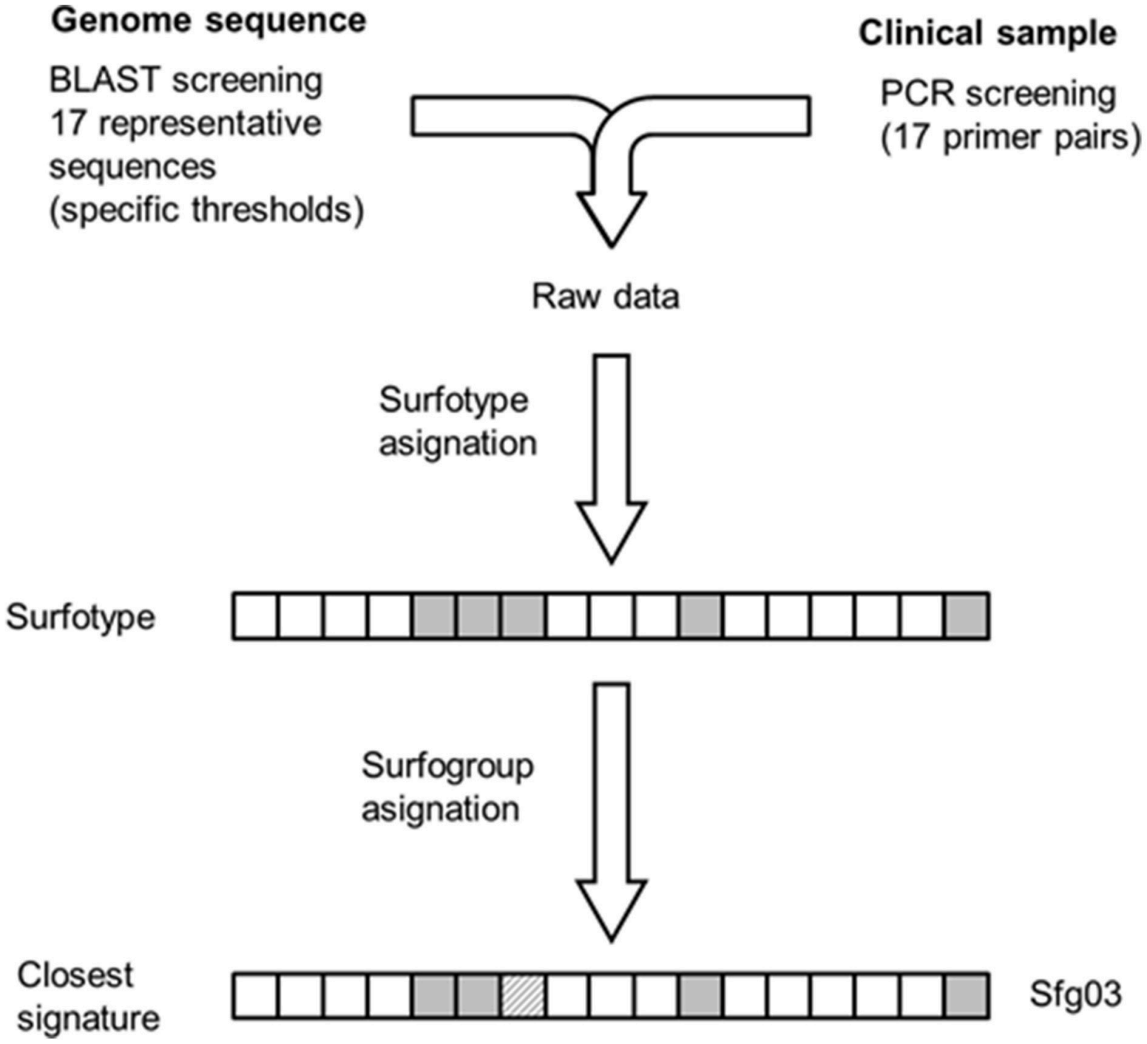
**Methodological scheme for surfotype and surfogroup assignment of test isolates**. Raw data derived from either sequencing or PCR is processed into a 17-mer Boolean vector (presence-full or absence-truncation). Assignment of surfotypes to the surfogroups showed in Figure 2 can be done through feature-by-feature comparison against the surfogroup signatures.

In this study, we have developed a strategy for formally classifying *S. pneumoniae* using the binary patterns of 17 highly discriminatory outer proteins (Figure 4). This allows for addressing the following issues: (1) to what extent outer protein profiles correlate to the invasive potential of pneumococcal clones and, consequently, the potential diagnostic applications of surfotypes; and (2) the relationship between the evolution of the surface proteome and the MLST genes. Despite what other similar studies have been reported (Dagerhamn et al., 2008; Desa et al., 2008; Imai et al., 2011; Browall et al., 2013), our approach is more comprehensive in terms of strain disparity, is focused on accessory surface proteins, and applies new statistical strategies. Surfotyping relies on profiles acquired via PCR screening or genomic sequencing, techniques which may lead to misleading results. Oligonucleotides may not anneal with sufficient affinity to template DNA in the case of a mismatch. Likewise, ORFs targeted in draft genomes might be interrupted by the contig limits and remain spuriously undetected. Nevertheless, these two methodologies complemented each other reasonably well. As illustrative examples, SG1-ST306 isolates, which cause invasive disease in young adults without prior colonization of the nasopharynx, were assigned to the primary invasive surfogroup Sfg06. 15A-ST63 clones, which typically cause acute exacerbations in COPD patients (Domenech et al., 2014), were classified as non-invasive Sfg10. The most remarkable exception was SG3-ST180, which was predicted to be an invasive opportunistic isolate after surfotyping despite being in a non-invasive position. This may be a consequence of the especially thick capsule of type 3, which would affect the activity of some protein determinants and therefore cause misclassification.

Despite the fact that SG-ST combinations are associated with different capacities to colonize human body niches and distinct patient types, current studies have failed to attribute virulent behavior to a single gene (Manso et al., 2014). Moreover, the contribution of a given gene to virulence seems dependent on other genome regions (Thomas et al., 2011). This is probably because the factors necessary for virulence are relatively redundant (Blomberg et al., 2009). There is evidence to support the idea that pneumococcal virulence is network-based and, therefore, a matter to be understood through the lens of systems biology, as proposed for *Staphylococcus aureus* (Sanchez et al., 2011). These pathofunctional networks may operate by following an orchestrated spatiotemporal pattern that eventually leads to a given clinical outcome. However, inferring explicit relationships between these proteins and disease is far from trivial considering that some of them play unknown or several roles. For instance, CbpG is not only involved in adherence to epithelium, but also in the cleavage of extracellular matrix (Mann et al., 2006). The non-invasive Sfg10 signature contains the sialic acid epimerase NanE, the putative Zn-scavenger PhtA (Rioux et al., 2011), and ZmpC, which prevents the influx of neutrophils (Surewaard et al., 2013). These three functions combined may favor long-term mucosae disease patterns and be selected for in isolates causing non-bacteriemic pneumonia and COPD acute exacerbations. The RrgA and StrD proteins, which are involved in the constitution and location of the adhesive pilus, are present in the opportunistic invasive surfogroups Sfg11, Sfg12, and Sfg15. This observation suggests that many of the discriminatory proteins selected in this work may be involved in long-term persistence and asymptomatic colonization. These processes have to be maintained until the infection is favored by particular host conditions. In this light, Sfg02, Sfg03, and Sfg04 surfogroups harbor the lowest number of surface proteins in the dataset, even though they are related to a primary invasive phenotype. Isolates belonging to these surfogroups have short colonization periods, and therefore would require less adhesive factors.

A relevant factor that could interfere to surfotyping is the introduction of pneumococcal conjugate vaccines. Some degree of co-evolution between the gene pools encoding the capsule and the accessory surfome could be expected, which together may largely determine the pathogenic behavior of a given pneumococcal lineage. In this light, the detection of surfogroups in capsular types in which they were not previously reported may be synonym of potential emergent clone generated by capsule switching and should be tracked.

MLST and surfotyping methods are conceptually different (Table 2). MLST genes evolve at a slow pace, making them appropriate for reconstructing the phylogeny of the species. MLST is based on the analysis of SNPs, which should not have a noticeable influence on protein function. In contrast, surfotyping prioritizes functions, which are subjected to strong selective pressure in terms of adaptation to defined pathogenic scenarios. Thus, surfotyping may be instrumental in detecting brusque genetic changes that could lead to emerging, highly-virulent clones. The 116 non-redundant surfotypes found describe a continuous 17th dimensional space, in which surfogroups can be observed as dense zones enriched in strains causing similar diseases. Genes that do not contribute sufficiently to the life-style phenotype may eventually be lost. This would explain why these genes are therefore absent from the signature. Surfogrouping merge some clonal complexes. However, large clonal complexes can be located in separate surfogroups. In particular, the giant CC156 surfogroup, whose size is a consequence of eBURST clustering collapse, reached a mere 40% surfogroup homogeneity. Meanwhile, the CC156 lineages derived from MLST-96 technique (Moschioni et al., 2013) are reasonably associated with different surfogroups.

**Table 2:** **Essential differences between MLST and Surfotyping**.

**Topic**	**MLST**	**Surfotyping**
Number of genes	Fixed (seven)	Variable (species-specific)
Type of variability	SNPs	Presence/absence, distant allelic variants (many residue changes), large insertions/deletions, mosaicism
Gene evolution rate	Slow	Fast
Protein location	Cytoplasm	Cell wall
Protein role	Housekeeping	Virulence
Correlation to pathogenity	Indirect association	Direct causality
Protein structural nature	Globular	Disordered regions, tandem repeats, anchor modules
Gene phyletic dispersion	Universal	Species-specific

Our results support a model of a complex association between pneumococcal surface factors and disease. The pathogen-host interaction would not behave according to a lock-and-key paradigm respect to their target molecules but as a bunch of keys for an array of locks. Pneumococci use its highly recombinogenic capacity as if they were “slot machines” whose winning feature combinations provide a higher efficiency for a given virulent scenario. This work provides a first report of the combinations that may be useful for predicting disease progression.

## Ethical standards

Written or oral informed consent was not required, because the source of the bacterial isolates was anonymized.

## Author contributions

AD and JM carried out the experimental work. CA, JL, AD, and AM designed and analyzed the assays. AM carried out the computational work and wrote the manuscript.

## Funding

This work was supported by a Miguel Servet contract from the Spanish Ministry of Health to AM, Plan Nacional de I+D+I of the Ministry of Science and Innovation (BIO2011-25343, BIO2014-555462-R, SAF2012-39444-C02), Fondo de Investigaciones Sanitarias de la Seguridad Social (PI11/00763) and Fondo Europeo de Desarrollo Regional (FEDER). CIBER Enfermedades Respiratorias is an initiative of the Instituto de Salud Carlos III.

### Conflict of interest statement

The authors declare that the research was conducted in the absence of any commercial or financial relationships that could be construed as a potential conflict of interest.
