# Supplementary material for: A Novel Typing Method for Streptococcus pneumoniae Using Selected Surface Proteins
Source: Front Microbiol. 2016 Mar 31;7:420. doi: 10.3389/fmicb.2016.00420 (PMC4815138; doi:10.3389/fmicb.2016.00420)
Supplement: Supplementary file 7 [file DataSheet7.docx]

| ***Surfogroup*** | ***Mean CC*** | ***Secondary CCs*** | ***Type of virulence*** | ***Reference*** | ***Commonest serotype*** |
| --- | --- | --- | --- | --- | --- |
| Sfg01 | CC156a | CC180, CC251, CC320, CC395 | Opportunistic invasive | (Lambertsen et al., 2010) | 9V |
| Sfg02 | CC180 | CC191 | Primary invasive | (Croucher et al., 2013) | 3 |
| Sfg03 | CC217 | CC242, CC2296 | Primary invasive | (Brueggemann et al., 2003,Syk et al., 2014) | 1 |
| Sfg04 | CC460 | CC191, CC242,CC395,CC1381,CC1390 | Primary invasive | (Pichon et al., 2013) | 6C |
| Sfg05 | CC1379 | CC306 | Primary invasive | (Bratcher et al., 2011,Janoir et al., 2014) | 6A/6C |
| Sfg06 | CC306 | None | Primary invasive | (Brueggemann et al., 2003) | 1 |
| Sfg07 | CC62 | CC235, CC306 | Non-invasive | (Brueggemann et al., 2003) | 11A |
| Sfg08 | CC156b | None | Primary invasive^a^ | None |  |
| Sfg09 | CC218 | CC156, CC1797 | Primary invasive | (Rakov et al., 2011) | 12F |
| Sfg10 | CC63 | CC62, CC100, CC156 | Non-invasive | (Nakano et al., 2016) | 15A |
| Sfg11 | CC66 | CC205 | Opportunistic invasive | (Wyres et al., 2013) | 9N,14,19F |
| Sfg12 | CC320 | CC558 | Opportunistic invasive | (Hsieh et al., 2013) | 19A |
| Sfg13 | CC199 | None | Opportunistic invasive | (Thomas et al., 2011,Vestrheim et al., 2012) | 19A |
| Sfg14 | CC15 | CC433 | Opportunistic invasive | (Lambertsen et al., 2010) | 14/19F |
| Sfg15 | CC156c | CC113, CC1536, CC2090 | Opportunistic invasive | None |  |
| Sfg16 | CC2090 | CC156c | Opportunistic invasive | (Zahner et al., 2010) | 19A |
| Sfg17 | CC81 | CC15 | Non-invasive | (Ma et al., 2013) | 23F |
| Sfg18 | CC439 | CC81 | Non-invasive | (Amrine-Madsen et al., 2008) | 6A/23F |

^a^ No references were available. Type of virulence was taken from the closest surfogroup (Sfg09)

**References**

1. Lambertsen, L., Brendstrup, M., Friis, H., Christensen, J. J. Molecular characterization of invasive penicillin non-susceptible *Streptococcus pneumoniae* from Denmark, 2001 to 2005. *Scand J Infect Dis* 2010; 42**:** 333-340.

2. Croucher, N. J., Mitchell, A. M., Gould, K. A., Inverarity, D., Barquist, L., Feltwell, T. et al. Dominant role of nucleotide substitution in the diversification of serotype 3 pneumococci over decades and during a single infection. *PLoS Genet* 2013; 9**:** e1003868.

3. Brueggemann, A. B., Griffiths, D. T., Meats, E., Peto, T., Crook, D. W., Spratt, B. G. Clonal relationships between invasive and carriage *Streptococcus pneumoniae* and serotype- and clone-specific differences in invasive disease potential. *J Infect Dis* 2003; 187**:** 1424-1432.

4. Syk, A., Norman, M., Fernebro, J., Gallotta, M., Farmand, S., Sandgren, A. et al. Emergence of hypervirulent mutants resistant to early clearance during systemic serotype 1 pneumococcal infection in mice and humans. *J Infect Dis* 2014; 210**:** 4-13.

5. Pichon, B., Ladhani, S. N., Slack, M. P., Segonds-Pichon, A., Andrews, N. J., Waight, P. A. et al. Changes in molecular epidemiology of *Streptococcus pneumoniae* causing meningitis following introduction of pneumococcal conjugate vaccination in England and Wales. *J Clin Microbiol* 2013; 51**:** 820-827.

6. Bratcher, P. E., Park, I. H., Oliver, M. B., Hortal, M., Camilli, R., Hollingshead, S. K. et al. Evolution of the capsular gene locus of *Streptococcus pneumoniae* serogroup 6. *Microbiology* 2011; 157**:** 189-198.

7. Janoir, C., Cohen, R., Levy, C., Bingen, E., Lepoutre, A., Gutmann, L. et al. Clonal expansion of the macrolide resistant ST386 within pneumococcal serotype 6C in France. *PLoS One* 2014; 9**:** e90935.

8. Rakov, A. V., Ubukata, K., Robinson, D. A. Population structure of hyperinvasive serotype 12F, clonal complex 218 *Streptococcus pneumoniae* revealed by multilocus boxB sequence typing. *Infect Genet Evol* 2011; 11**:** 1929-1939.

9. Nakano, S., Fujisawa, T., Ito, Y., Chang, B., Suga, S., Noguchi, T. et al. Serotypes, antimicrobial susceptibility, and molecular epidemiology of invasive and non-invasive *Streptococcus pneumoniae* isolates in paediatric patients after the introduction of 13-valent conjugate vaccine in a nationwide surveillance study conducted in Japan in 2012-2014. *Vaccine* 2016; 34**:** 67-76.

10. Wyres, K. L., Lambertsen, L. M., Croucher, N. J., McGee, L., von, G. A., Linares, J. et al. Pneumococcal capsular switching: a historical perspective. *J Infect Dis* 2013; 207**:** 439-449.

11. Hsieh, Y. C., Lin, T. L., Chang, K. Y., Huang, Y. C., Chen, C. J., Lin, T. Y. et al. Expansion and evolution of *Streptococcus pneumoniae* serotype 19A ST320 clone as compared to its ancestral clone, Taiwan19F-14 (ST236). *J Infect Dis* 2013; 208**:** 203-210.

12. Thomas, J. C., Figueira, M., Fennie, K. P., Laufer, A. S., Kong, Y., Pichichero, M. E. et al. *Streptococcus pneumoniae* clonal complex 199: genetic diversity and tissue-specific virulence. *PLoS One* 2011; 6**:** e18649.

13. Vestrheim, D. F., Steinbakk, M., Aaberge, I. S., Caugant, D. A. Postvaccination increase in serotype 19A pneumococcal disease in Norway is driven by expansion of penicillin-susceptible strains of the ST199 complex. *Clin Vaccine Immunol* 2012; 19**:** 443-445.

14. Zahner, D., Gudlavalleti, A., Stephens, D. S. Increase in pilus islet 2-encoded pili among Streptococcus pneumoniae isolates, Atlanta, Georgia, USA. *Emerg Infect Dis* 2010; 16**:** 955-962.

15. Ma, X., Yao, K. H., Yu, S. J., Zhou, L., Li, Q. H., Shi, W. et al. Genotype replacement within serotype 23F *Streptococcus pneumoniae* in Beijing, China: characterization of serotype 23F. *Epidemiol Infect* 2013; 141**:** 1690-1696.

16. Amrine-Madsen, H., Van, E. J., Mera, R. M., Miller, L. A., Poupard, J. A., Thomas, E. S. et al. Temporal and spatial distribution of clonal complexes of *Streptococcus pneumoniae* isolates resistant to multiple classes of antibiotics in Belgium, 1997 to 2004. *Antimicrob Agents Chemother* 2008; 52**:** 3216-3220.
