# Supplementary material for: A Novel Typing Method for Streptococcus pneumoniae Using Selected Surface Proteins
Source: Front Microbiol. 2016 Mar 31;7:420. doi: 10.3389/fmicb.2016.00420 (PMC4815138; doi:10.3389/fmicb.2016.00420)
Supplement: Supplementary file 9 [file Image1.PDF]

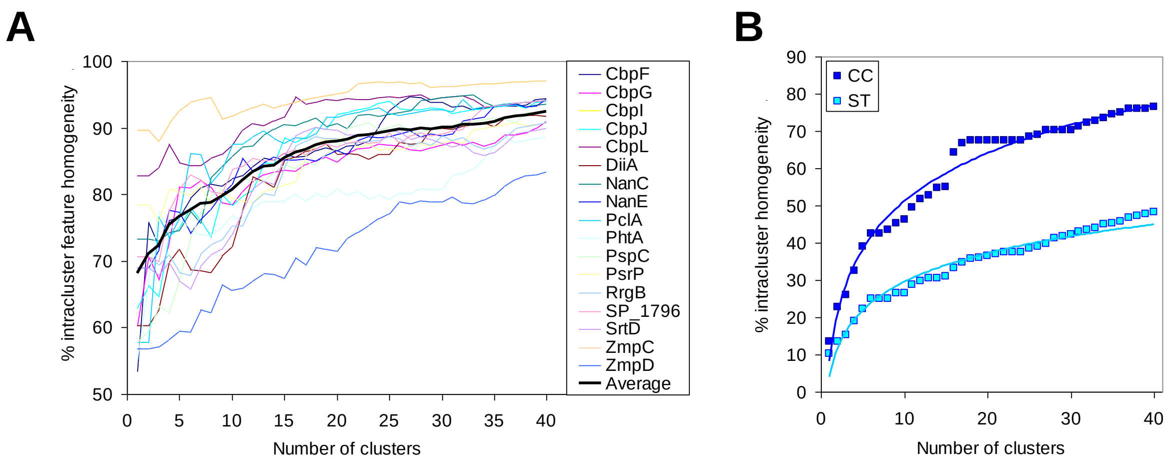

Figure S1. Cluster validation by quality estimators. Average intracluster NC (A) and clonal complex/ST homogeneity (B) where a range of 1-40 cluster granularity were evaluated. NC in a given cluster ranged from 50% (null hypothesis, no correlation between features and clusters) and 100% (perfect correlation).
