# Supplementary material for: A Novel Typing Method for Streptococcus pneumoniae Using Selected Surface Proteins
Source: Front Microbiol. 2016 Mar 31;7:420. doi: 10.3389/fmicb.2016.00420 (PMC4815138; doi:10.3389/fmicb.2016.00420)
Supplement: Supplementary file 10 [file Image2.PDF]

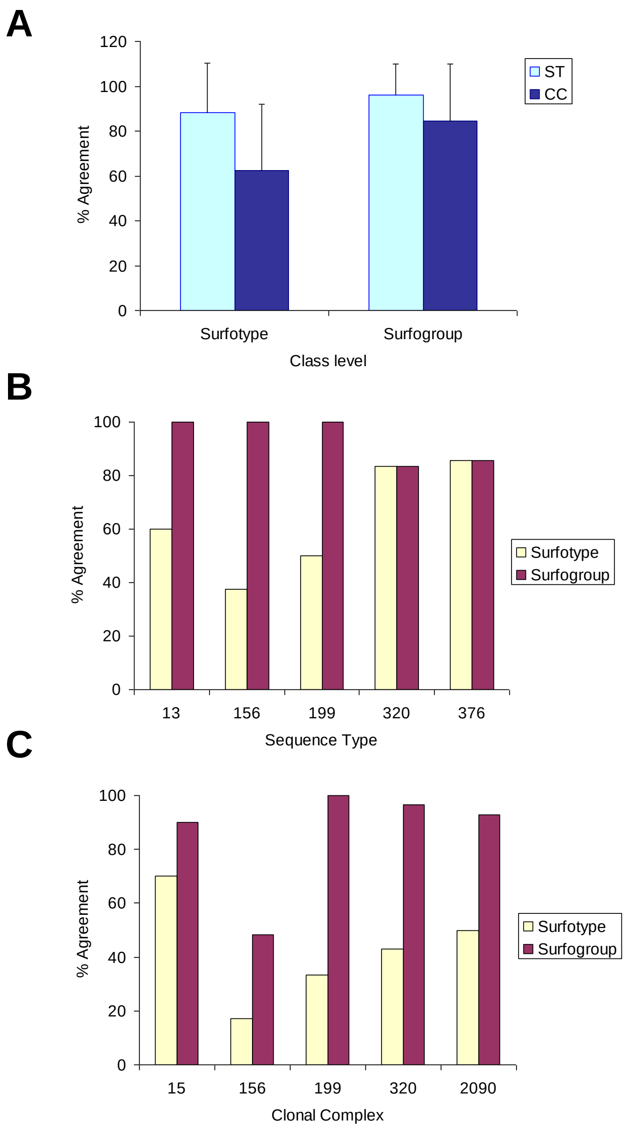

Figure S2. Homogeneity of surfotyping and surfogrouping within ST and clonal complexes. A. MLST agreement at surfotype and surfogroup levels. B. Intraclonal surfomic agreement of the most prominent STs. C. Intraclonal surfomic agreement of the most prominent CCs. Allele sequences, ST combinations and clonal complex founders were downloaded from http://spneumoniae.mlst.net.
